# Clinical Complexity in Atrial Fibrillation: A Call for a Multidimensional Framework

**DOI:** 10.1016/j.jacadv.2026.102874

**Published:** 2026-06-10

**Authors:** Giulio Francesco Romiti, Gregory Y.H. Lip, Marco Proietti

**Affiliations:** a Department of Wellbeing, Health and Environmental Sustainability, Sapienza–University of Rome, Rieti, Italy; b Institute of Life Course and Medical Sciences, University of Liverpool, Liverpool, United Kingdom; c Danish Center for Health Services Research, Department of Clinical Medicine, Aalborg University, Aalborg, Denmark; d Department of Cardiology, Lipidology and Internal Medicine with Intensive Coronary Care Unit, Medical University of Bialystok, Bialystok, Poland; e Department of Clinical Sciences and Community Health, University of Milan, Milan, Italy; f Division of Cardiogeriatric Subacute Care, IRCCS Istituti Clinici Scientifici Maugeri, Milan, Italy

Over the past few decades, the epidemiology of atrial fibrillation (AF) has changed substantially. Patients with AF are older, with an increasing burden of frailty,^1^ multimorbidity^2^ (with consequential polypharmacy), and growing exposure to adverse social determinants of health (SDOH),^3^ with profound impacts on clinical trajectories. These factors rarely occur in isolation in patients with AF; rather, they interact at the individual level, and their coexistence poses substantial challenges to the clinical management.

This observation is common ground among physicians who care for AF patients in their daily practice; however, research in this area has usually focused on each of these determinants individually, failing to offer a conceptual framework for capturing the true underlying complexity of these patients. The lack of such a unitary conceptual construct translates into an incomplete response to their health needs. Indeed, current guidelines recommend integrated approaches to manage AF, which nevertheless place predominant emphasis on the management of comorbidities.^4^^,^^5^ Moreover, although these approaches offer a framework for clinical management, they are not intended to serve as conceptual models through which the interaction between these determinants of health can be understood. A unitary framework is needed and is the first, preliminary step toward designing the next generation of integrated, multidimensional care models, to provide comprehensive answers to the growing health needs of these patients.

Conceptualizations of patient complexity have been previously proposed to capture the interaction between different determinants of health^6^^,^^7^ and to describe patients whose needs exceed those represented by single-disease models. This approach appears particularly well suited for translation to current AF patients, for whom operationalization has been previously performed.^8^ Here, we propose *clinical complexity* as a multidimensional framework for understanding how the different determinants of health co-occur and interact in contemporary patients with AF. In the context of AF, clinical complexity unfolds along 5 constitutive domains—biological, disease-related, functional, health care–induced, and contextual—that interact at the individual level and influence disease trajectories, through pathways that no single determinant can capture comprehensively (Figure 1). Recognizing clinical complexity as a construct aligns AF care with the multifaceted health needs of patients, which require multidimensional responses that have yet to be articulated.

**Figure 1:**
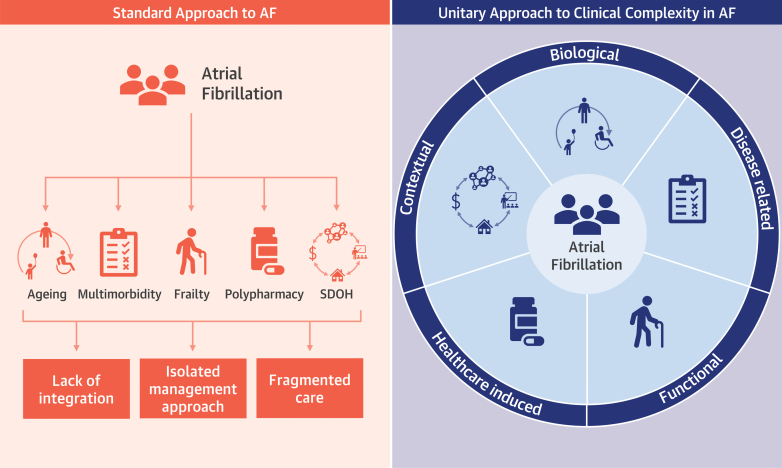
Clinical Complexity in Patients With Atrial Fibrillation Although each determinant of health is conventionally studied in isolation, *clinical complexity* considers their interaction along 5 constitutive domains that influence the trajectory of contemporary patients with AF. AF = atrial fibrillation; SDOH = social determinants of health.

## Constitutive domains of clinical complexity

Each constitutive domain of clinical complexity is characterized by hallmark determinants, which represent its most common expression in patients with AF. Yet, these domains are entwined, and it is their interaction that drives clinical trajectories.

The biological domain encompasses the individual substrate on which AF unfolds and is managed. Chronological aging is its hallmark expression, driving AF incidence and prognosis,^5^ and providing the substrate along which the other determinants accumulate; sex differences and genetics contribute further to this dimension.

The disease-related domain encompasses the contribution of comorbidities carried by the patient. Multimorbidity (usually defined as the co-existence of ≥2 conditions) is its hallmark determinant, but its impact on AF prognosis exceeds the simple accumulation of diseases: individual comorbidities (such as prior stroke, history of bleeding, chronic kidney disease, and cancer) have a disproportionate impact on AF management; moreover, diseases tend to cluster in nonrandom patterns, which exert a synergistic (rather than merely additive) effect on prognosis.

The functional domain captures physiological reserve, autonomy, and resilience to stressors. Its hallmark expression is frailty, which can be found in up to 40% of patients with AF, and largely drives morbidity and mortality.^1^ The functional domain is conceptually distinct from the disease-related one (and so is frailty from multimorbidity), in that—unlike most diseases—it focuses on decline of functions, and is potentially reversible. More recently, the concept of intrinsic capacity (defined as the composite of an individual's physical and mental capacities) has emerged as complementary to frailty, although its implementation in practice remains challenging.^9^

The health care–induced domain encompasses the contribution of the health care system to individual patient complexity, and polypharmacy (ie, the simultaneous use of ≥5 drugs in a single patient) is its hallmark manifestation. Although multimorbidity unavoidably leads to polypharmacy, inappropriate polypharmacy occurs when treatments lack strong clinical indications or are used irrespective of patient-specific factors that may alter their risk/benefit profile. Inappropriate polypharmacy has detrimental effects on adherence, side effects, and risk of drug interactions^10^ and is often caused by fragmentation of care, which is another expression of the health care–induced domain of clinical complexity.

The contextual domain encompasses the contribution of social, lifestyle, and environmental determinants to clinical complexity. Lifestyle factors and adverse SDOH are the most studied expression of this domain,^3^, ^4^, ^5^ although the contribution of the exposome to the clinical complexity of patients with AF has more recently emerged. The contextual domain exerts its effect along the entire AF trajectory—from diagnosis to treatment, access to care, and outcomes.

## Implications for practice and research

Defining constitutive domains is useful to identify the main pillars of clinical complexity in patients with AF; however, at individual level, their joint contribution is inextricable. For example, fragmentation of care is a pivotal expression of the health care–induced domain of clinical complexity, but at individual level it is fostered by adverse SDOH and inequities in access to care; heart failure is a common comorbidity in patients with AF, but the decline in functions and autonomy it may cause pertains to the functional domain. Clinical complexity offers a lens that captures the contribution of these interacting determinants in ways that neither single-dimension construct nor their simple “sum” can represent.

Introducing clinical complexity as a construct has 3 practical implications. First, it allows a more comprehensive identification of the patient health needs, which are not limited to single determinants (eg, comorbidities), and cannot find appropriate answers from the standard, siloed models of care. Second, it provides the conceptual substrate for AF-clinic models built around multidisciplinarity, incorporating patients’ preferences and values, and truly empowering shared decision-making, where appropriateness and proportionality of treatment decisions are at the core of management. Third, conceptualizing clinical complexity is the first, necessary step for the development of a modern research agenda that focuses on the joint contribution of determinants, rather than their isolated effects, allowing for the development of dedicated assessment tools and management strategies that advance further the care of patients with AF.

The epidemiology of AF is rapidly evolving, and health care systems must adapt to manage the growing health needs of these patients. We believe that adopting clinical complexity as a unitary, multidimensional framework—subject to the revision any working construct requires—is a crucial first step needed to advance the management of contemporary patients with AF, and to align research to their health demands.

## Funding support and author disclosures

Dr Romiti is supported by a grant issued by Sapienza–University of Rome (B83C26001140005); has been a consultant for Boehringer Ingelheim; and has received an educational grant from Anthos (no fees were directly received personally). Dr Lip has performed institutional consultancy and/or speaker roles for BMS/Pfizer, Boehringer Ingelheim, Anthos, and Huawei (no fees were received personally; all the disclosures happened outside of the submitted work); he is a National Institute for Health and Care Research (NIHR) Senior Investigator Emeritus and co-PI of the AFFIRMO project on multimorbidity in AF (grant agreement number 899871), TARGET project on digital twins for personalized management of atrial fibrillation and stroke (grant agreement number 101136244), and ARISTOTELES project on artificial intelligence for management of chronic long term conditions (grant agreement number 101080189), which are all funded by the EU’s Horizon Europe Research & Innovation program. Dr Proietti has been a speaker for Pfizer and BMS/JJ Alliance; has been a consultant for Regeneron Pharmaceuticals; and he is Italian National Principal Investigator of the AFFIRMO project on multimorbidity in atrial fibrillation, which has received funding from the European Union’s Horizon 2020 research and innovation program under grant agreement number 899871.
